# Longitudinal progression of choroid plexus enlargement is associated with female sex, cognitive decline and ApoE E4 homozygote status

**DOI:** 10.3389/fpsyt.2023.1039239

**Published:** 2023-03-08

**Authors:** Julie Novakova Martinkova, Maria Teresa Ferretti, Alberto Ferrari, Ondrej Lerch, Veronika Matuskova, Juraj Secnik, Jakub Hort, for the Alzheimer’s Disease Neuroimaging Initiative

**Affiliations:** ^1^Cognitive Center, Department of Neurology, Second Faculty of Medicine, Charles University and Motol University Hospital, Prague, Czech Republic; ^2^Women’s Brain Project, Gunterhausen, Switzerland; ^3^Center for Alzheimer Research, Division of Clinical Geriatrics, Department of Neurobiology, Care Sciences and Society, Karolinska Institutet, Huddinge, Sweden

**Keywords:** choroid plexus, longitudinal analysis, sex differences, Alzheimer’s disease, cognitive impairment

## Abstract

**Introduction:**

Choroid plexus (CP)-related mechanisms have been implicated in the pathogenesis of neurodegenerative diseases, including Alzheimer’s disease. In this pilot study, we aimed to elucidate the association between longitudinal changes in CP volume, sex and cognitive impairment.

**Methods:**

We assessed longitudinal changes in CP volume in a cohort of *n* = 613 subjects across *n* = 2,334 datapoints from ADNI 2 and ADNI-GO, belonging to cognitively unimpaired (CN), stable mild cognitive impairment (MCI), clinically diagnosed Alzheimer’s disease dementia (AD) or convertor (to either AD or MCI) subgroups. CP volume was automatically segmented and used as a response variable in linear mixed effect models with random intercept clustered by patient identity. Temporal effects of select variables were assessed by interactions and subgroup analyses.

**Results:**

We found an overall significant increase of CP volume in time (14.92 mm^3^ per year, 95% confidence interval, CI (11.05, 18.77), *p* < 0.001). Sex-disaggregated results showed an annual rate of increase 9.48 mm^3^ in males [95% CI (4.08, 14.87), *p* < 0.001], and 20.43 mm^3^ in females [95% CI (14.91, 25.93), *p* < 0.001], indicating more than double the rate of increase in females, which appeared independent of other temporal variables. The only diagnostic group with a significant CP increase as compared to CN was the convertors group, with an increase of 24.88 mm^3^/year [95% CI (14, 35.82), *p* < 0.001]. ApoE exhibited a significant temporal effect, with the E4 homozygote group’s CP increasing at more than triple the rate of non-carrier or heterozygote groups [40.72, 95% CI (25.97, 55.46), *p* < 0.001 vs. 12.52, 95% CI (8.02, 17.02), *p* < 0.001 for ApoE E4 homozygotes and E4 non-carriers, respectively], and may have modified the diagnostic group relationship.

**Conclusion:**

Our results contribute to potential mechanisms for sex differences in cognitive impairment with a novel finding of twice the annual choroid plexus enlargement in females and provide putative support for CP-related mechanisms of cognitive deterioration and its relationship to ApoE E4.

## 1. Introduction

Choroid plexus (CP) is a highly vascularized epithelial structure located in the ventricles with several crucial functions for maintaining cerebral homeostasis. In addition to producing cerebrospinal fluid (CSF) and forming the blood-CSF barrier (1), CP excretes numerous compounds implicated in neurogenesis (2), growth factor production (3), and is considered a key structure for initiating cerebral inflammatory responses (4–8).

Inflammation and CP alterations have been increasingly recognized both in normal ageing and neurodegenerative diseases such as Alzheimer’s disease (AD) (9). CP pro-inflammatory signaling was found to be up-regulated (10, 11), and associated with beta-amyloid accumulation (12) as well as sex hormones (13). CP contains specific beta-amyloid scavengers, whose decline has been linked to increased cerebral beta-amyloid accumulation, including in the CP, with further perpetuation by CP and blood-CSF barrier failure (14–16). Interestingly, the expression of these scavengers in animal models was found to be sex-dependent, with lower levels in females (17). Sex is also an important driver of immunologic heterogeneity both in general (18, 19), and specifically in neurodegeneration (20–23), suggesting that known sex differences in AD (24) could be related to CP pathology.

Since directly establishing human CP function and composition *in vivo* is challenging, imaging approaches, including volumetry, are progressively recognized. CP enlargement has been linked with neuroinflammation in multiple sclerosis in both animals and human subjects (7). An increase in CP volume has been noted in ageing (25), and disease states linked with inflammation, such as complex regional pain (26), psychosis and schizophrenia (27, 28), depression (29), stroke (30), multiple sclerosis (31–33), obesity (34), and cross-sectionally in AD (35–37). Choroid plexus-mediated mechanisms represent potential promising targets for future treatments based on animal models of AD (38).

Sex differences in CP volume have, to our knowledge, been assessed only cross-sectionally: in healthy subjects (25) or multiple sclerosis (31), no difference was found, and in Alzheimer’s disease (37), males were noted to have larger CP volumes than females. However, cross-sectional analysis of volumetric sex differences is complicated by the need for precise intracranial volume correction, which is often the source of false-positives and inconsistencies (39). Within-subject longitudinal comparison eliminates the inter-subject intracranial volume variability.

In addition, longitudinal follow-up enables uncovering potential causal relationships due to specifically associating temporal changes with exposure to disease-related changes and progression. Further exploration of longitudinal changes in CP is warranted due to its potential role in AD pathophysiology and as an AD treatment target. Exploring potential sex differences can provide more insight into AD heterogeneity and sex-specific pathophysiologic and prognostic factors. To our knowledge, longitudinal changes in CP volume, their association with cognitive impairment progression or potential sex differences have not been explored.

In the present study, we retrospectively investigated volumetric CP changes in a large longitudinally followed cohort (*n* = 613 subjects across *n* = 2,334 datapoints) comprising patients across the spectrum of cognitive impairment. We aimed to disentangle potential relationships between cognitive impairment, sex and the choroid plexus, specifically: (1) investigate sex differences in longitudinal changes in choroid plexus volume, (2) assess temporal relationships of the choroid plexus and three additional variables of interest: diagnostic group, age at baseline and ApoE E4 allele number.

## 2. Methods

### 2.1. Study design and population

We investigated CP volume in a cohort including patients with no impairment (CN, cognitively normal), mild cognitive impairment (MCI) or clinically diagnosed Alzheimer’s disease dementia (AD) from the Alzheimer’s Disease Neuroimaging Initiative (ADNI) dataset. ADNI was launched in 2003 as a public-private partnership, led by Principal Investigator Michael W. Weiner, MD. The primary goal of ADNI has been to test whether serial magnetic resonance imaging (MRI), positron emission tomography (PET), other biological markers, and clinical and neuropsychological assessment can be combined to measure the progression of mild cognitive impairment (MCI) and early Alzheimer’s disease (AD). Data used in the preparation of this article were obtained from the ADNI database (adni.loni.usc.edu).

ADNI2 defines CN subjects as free of memory complaints at baseline, with scores between 24 and 30 on the Mini Mental State Exam (MMSE), normal scores from the Wechsler logical memory education-adjusted scale, and Clinical Dementia Rating (CDR) of 0. MCI subjects exhibit a subjective memory complaint, MMSE 24–30, CDR 0.5 and abnormal Wechsler logical memory scores, while not fulfilling the criteria for a dementia diagnosis. AD diagnosis is made based on subjective memory complaints, MMSE scores of 20–26 at baseline, CDR 0.5–1.0, and following the NINCDS/ADRDA criteria for probable AD (40). All patients with other significant neurologic/psychiatric disorders or suspicion of non-AD pathology are excluded, as detailed in the full protocol (41).

According to previous reports, the majority of ADNI study population falls into the 55–90 age range at baseline. Most subjects are at least moderately educated, with high proportions of university-educated participants (42). This was also true for our study, as reported in section 3.1 and Table 1A.

**Table 1:** Study population overview overall and by sex **(A)** and by diagnostic group **(B)**.

**(A)**						
		**Overall**	**Female**		**Male**	***p***
*n*		613	295		318	
Diagnostic group (%)	CN	188 (30.7)	105 (35.6)		83 (26.1)	0.086
	MCI	239 (39.0)	108 (36.6)		131 (41.2)	
	AD	88 (14.4)	38 (12.9)		50 (15.7)	
	Convert	98 (16.0)	44 (14.9)		54 (17.0)	
Age at baseline [Y] (mean (SD))		72.69 (7.25)	71.43 (7.10)		73.86 (7.21)	<0.001
No. of visits (median [IQR])		4.00 [3.00, 5.00]	4.00 [3.00, 5.00]		4.00 [3.00, 5.00]	0.728
Total follow-up length [Y] (median [IQR])		2.10 [1.21, 4.01]	2.09 [1.19, 4.01]		2.10 [1.21, 4.00]	0.908
Mean time between visits [Y] (median [IQR])		0.77 [0.67, 1.03]	0.78 [0.67, 1.02]		0.77 [0.67, 1.03]	0.892
ApoE e4 allele [No] (%)	0	323 (52.7)	151 (51.2)		172 (54.1)	0.195
	1	228 (37.2)	119 (40.3)		109 (34.3)	
	2	62 (10.1)	25 (8.5)		37 (11.6)	
Education [Y] (median [IQR])		16.00 [14.00, 18.00]	16.00 [14.00, 18.00]		17.00 [16.00, 19.00]	<0.001
Average choroid plexus volume [mm^3^] (mean (SD))		2220.75 (434.34)	2020.55 (368.32)		2406.47 (408.05)	<0.001
Average lateral ventricles volume [mm^3^] (median [IQR])		16928.00 [11428.00, 23728.00]	14051.00 [8825.00, 19877.00]		19863.25 [14988.25, 27001.50]	<0.001
MMSE at baseline (median [IQR])		28.00 [26.00, 30.00]	29.00 [27.00, 30.00]		28.00 [26.00, 29.00]	0.004
Hypertension (%)	No	324 (52.9)	168 (56.9)		156 (49.1)	0.061
	Yes	289 (47.1)	127 (43.1)		162 (50.9)	
BMI at baseline (mean (SD))		27.50 (4.98)	27.54 (5.64)		27.46 (4.28)	0.839
GDS at baseline (median [IQR])		1.00 [0.00, 2.00]	1.00 [0.00, 2.00]		1.00 [0.00, 2.00]	0.926
**(B)**						
		**CN**	**MCI**	**AD**	**Convert**	***p***
*n*		188	239	88	98	
Sex (%)	Female	105 (55.9)	108 (45.2)	38 (43.2)	44 (44.9)	0.086
	Male	83 (44.1)	131 (54.8)	50 (56.8)	54 (55.1)	
Age at baseline [Y] (mean (SD))		72.62 (6.57)	71.88 (7.39)	74.09 (8.03)	73.53 (7.27)	0.055
No. of visits (median [IQR])		4.00 [3.00, 5.00]	4.00 [3.00, 5.00]	3.00 [3.00, 3.00]	4.00 [3.00, 5.00]	<0.001
Total follow-up length [Y] (median [IQR])		2.18 [2.03, 4.05]	2.16 [1.21, 4.03]	1.07 [1.02, 2.00]	2.14 [2.02, 4.04]	<0.001
Mean time between visits [Y] (median [IQR])		1.01 [0.70, 1.06]	0.76 [0.67, 1.02]	0.55 [0.52, 0.69]	0.79 [0.68, 1.02]	<0.001
ApoE e4 allele [No] (%)	0	130 (69.1)	137 (57.3)	20 (22.7)	36 (36.7)	<0.001
	1	54 (28.7)	82 (34.3)	46 (52.3)	46 (46.9)	
	2	4 (2.1)	20 (8.4)	22 (25.0)	16 (16.3)	
Education [Y] (median [IQR])		17.00 [16.00, 19.00]	16.00 [14.00, 18.00]	16.00 [14.00, 18.00]	16.00 [14.00, 18.00]	0.002
Average choroid plexus volume [mm^3^] (mean (SD))		2149.19 (379.38)	2182.41 (415.16)	2396.01 (466.34)	2294.14 (497.99)	<0.001
Average lateral ventricles volume [mm^3^] (median [IQR])		15223.00 [10301.62, 21045.62]	16954.50 [10449.00, 23693.50]	21745.50 [16274.62, 28269.38]	17353.50 [13038.62, 24547.00]	<0.001
MMSE at baseline (median [IQR])		29.00 [29.00, 30.00]	29.00 [27.00, 29.00]	23.00 [21.00, 25.00]	28.00 [26.00, 29.00]	<0.001
Hypertension (%)	0	103 (54.8)	123 (51.5)	50 (56.8)	48 (49.0)	0.657
	1	85 (45.2)	116 (48.5)	38 (43.2)	50 (51.0)	
BMI at baseline (mean (SD))		27.89 (5.38)	27.79 (4.75)	25.74 (4.73)	27.59 (4.65)	0.004
GDS at baseline (median [IQR])		0.00 [0.00, 1.00]	2.00 [1.00, 3.00]	1.00 [1.00, 2.00]	1.50 [1.00, 2.00]	<0.001

Values at baseline for all longitudinally recorded variables. Reported as *N* (%) for categorical variables, mean (standard deviation) for continuous normally distributed variables and median [interquartile range] for non-normally distributed variables. Statistical significance of mean difference between men and women/diagnostic groups was calculated using the Chi-square test (categorical variables), one-way analysis of variance (ANOVA) – continuous normally distributed variables, and the Kruskal–Wallis (continuous non-normal variables). CN, cognitively normal (stable or improved across follow-up); MCI, mild cognitive impairment (stable or improved across follow-up); AD, Alzheimer’s disease dementia (stable); convert, conversion to a more advanced impairment group (MCI or dementia) across follow-up; Y, years; SD, standard deviation; IQR, interquartile range; MMSE, mini mental state exam; BMI, body mass index; GDS, geriatric depression scale 15.

For the purpose of this study, we only included individuals from the ADNI2 or ADNI GO datasets with available high resolution 3T T1 structural MRIs, who were evaluated at least twice (to enable longitudinal analyses) with no missing data in main analyzed variables (choroid plexus volume, sex, age, ApoE, diagnostic group). All individuals with cognitive impairment due to clinically suspected non-Alzheimer’s pathology were excluded. Additionally, due to previous reports on increased CP in stroke patients (30), we have specifically excluded patients with a history of stroke.

Our study utilized data obtained from the ADNI data repository. All ADNI studies are conducted according to the Good Clinical Practice guidelines, the Declaration of Helsinki, and United States. Twenty-one CFR Part 50 (Protection of Human Subjects), and Part 56 (Institutional Review Boards). Written informed consent was obtained from all participants before protocol-specific procedures were performed. The ADNI protocol was approved by the Institutional Review Boards of all of the participating institutions.

### 2.2. Categorization by diagnosis

We assessed whether the subject’s diagnostic group changed during follow-up, and then categorized them as follows: stable no impairment (CN), stable MCI (MCI), stable AD (AD); those who progressed to a higher impairment group (whether MCI or AD) were grouped as “convertors,” while those whose diagnostic group improved were combined with their follow-up “stable” diagnosis due to the comparably insignificant size of the category.

### 2.3. Sex and gender

Sex refers to an individual’s biologic characteristic, determined by their anatomy, which is a product of sex chromosomes (XX, XY) and sex hormones. Gender is a social construct that arises from self-identification and interactions with the individual’s social environment. ADNI does not specify the method of patient sex/gender determination, we therefore opted to use “sex” since our study focuses on the biological characteristics of brain morphology.

### 2.4. MRI acquisition and analysis

The MRI images were acquired according to ADNI 2 protocol.^1^ The current study utilized CP volume and lateral ventricle (LV) volume measurements from the University of California, San Francisco (UCSF) longitudinal FreeSurfer datasets. Cortical reconstruction and volumetric segmentation of CP and lateral ventricles was performed by the FreeSurfer image analysis software,^2^ version 5.1. Images were automatically processed using the longitudinal stream (43) in FreeSurfer. The FreeSurfer method has been described elsewhere (44), in brief: manually labeled training data is used to construct a complex probabilistic atlas. Images are segmented by normalizing subject data to common space and applying the probabilistic atlas to image-specific voxel intensities, selecting the optimal segmentation. In this context, Freesurfer CP segmentation includes the full volume of CP in the lateral ventricles (including both cells and stroma). The FreeSurfer approach to CP segmentation has been validated by its use in several previous studies (25–28, 30, 34, 35). All images underwent quality control at Mayo Clinic, Rochester, and subsequent Freesurfer segmentation quality control at UCSF. Only those that passed the quality control for lateral ventricles (relevant for both ventricular volume and CP volume) were selected for this study.

Due to lack of preceding hypothesis about CP laterality, we used combined left and right CP volumes (averaged) as segmented by the Desikan-Killany atlas, similarly to previous studies (30, 35). To determine if CP volume change and its temporal relationships were comparable to ventricular changes, we utilized a combined measure of the segmented lateral ventricle (LV) a inferior LV volumes. We opted to only use LV volume (excluding the third and fourth ventricle volumes) to best match FreeSurfer CP segmentation limitations; this approach was similar to previous studies (30).

### 2.5. Statistical analysis

To calculate descriptive statistics, we stratified the dataset by sex and by diagnostic group and used the chi-square test, one-way analysis of variance (ANOVA) or the Kruskal–Wallis test to test for differences between subgroups as appropriate. Normality was assessed by visual inspection of histograms.

To determine the effect of time and other variables on CP volume, we fitted linear mixed effects models with random intercept using clustering by subject identificator, utilizing CP volume as the dependent variable. We replicated the main analyses in models with random intercept and slope; since we saw no meaningful change, we opted to use random intercept only for ease of interpretation.

Previous reports have highlighted the potential effects of arterial hypertension (37), depression (29), and body adiposity (34) on CP volumes. Thus, we included the following in our analysis: history of arterial hypertension, total result of the Geriatric Depression Scale 15 at baseline [GDS, a proxy of depressive symptoms, values equal to or larger than 5 are considered as possible depression (45)], and body mass index (BMI) at baseline, calculated as weight in kg/height in cm^2^. Where baseline values were not available, we used first available follow-up values.

First, we assessed temporal change in CP volume (dependent variable) in an overall pooled dataset in a simple unadjusted model, followed by a model adjusted for baseline effects of diagnostic group, sex, baseline age, ApoE, hypertension, GDS and BMI (independent variables). Next, we conducted subgroup analyses (using models adjusted for age, sex, ApoE/diagnostic group as appropriate) to quantify the effects of ApoE (ApoE e4 non-carriers, heterozygotes or homozygotes), diagnostic subgroups (CN, MCI, convertors or AD) and sex (male/female). In subsequent analyses, we assessed the temporal effects of the four main variables (diagnostic group, sex, ApoE and baseline age, independent variables) on CP volume (dependent variable), by using the time × variable interaction component to determine the significance of the temporal effects. Final models were adjusted for baseline effects of the three other independent variables of interest. Additionally, we have created three separate models with either arterial hypertension, GDS or BMI as independent variables, along with the four previous independent variables (diagnostic group, sex, baseline age, and ApoE), time, and time × variable interaction, and CP volume as the dependent variable.

We replicated the main analyses adjusting for baseline effects of education attainment and study site as independent variables; since the obtained results did not exhibit an appreciable difference, we subsequently excluded these variables.

Next, we aimed to disentangle temporal relationships between variables by including more complex interaction models. We repeated adjusted models as described above (adjusted for age, sex, ApoE or diagnostic group as appropriate), this time using two time × variable relationships for each combination of the four main variables, as well as their three-way interactions with time. Similar analyses were conducted for arterial hypertension, GDS and BMI, and their interactions with the four main variables.

To further clarify the association between CP volume in time and baseline age, as well as its relationship to sex, we assessed the time × age interaction (adjusted for baseline effects of diagnostic group and ApoE) in both male and female subgroups. We then separated baseline age into quartiles (delineated by the following ages: 55.0, 67.6, 72.1, 77.5, and 91.4), and calculated predicted CP volumes at each time increment of follow-up time in each of the quartiles for both sexes. To confirm the results, we also fitted separate models for each of the quartile subgroups (CP volume as dependent variable, independent variables: time, diagnostic group, ApoE, sex, sex × time interaction).

To assess if comparable effects were present in ventricular volume change, we also fitted the same basic models with combined LV volume as the dependent variable. We used log transformation on the LV volume to control its significant left skew. The temporal effect of LV was additionally tested against the CP as predictor to assess the CP × LV temporal relationships.

In addition, where this information was available, we checked whether head coil changes or MRI software changes from baseline (or earliest follow-up where this information was available) to last available follow-up occured. Neither exclusion of segmentations with a head coil change nor addition of a binary software change variable (or software change x time) appeared to influence the conclusions of the study.

We set the statistical significance threshold at 0.05. All analyses were conducted in R version 4.2.1 (46), using (among others) the packages lme4 (47), lmer test (48), and ggplot2 (49).

## 3. Results

### 3.1. Descriptive statistics

The current study included *N* = 613 subjects in total, of who 295 (48.1%) were women (Table 1A). Participants were followed for a median of 4 visits over 2.10 years (0.77 years between visits). Age at baseline had the following characteristics: median [interquartile range] was 72.11 [67.61, 77.48], with minimum 55.01 and maximum 91.43 years old.

Women were overall younger at baseline (71.43 years baseline age for females, on average, and 73.86 for males, *p* < 0.001) and were more represented in the CN group (55.9%) than in other diagnostic groups (43.2–45.2%), with no significant sex difference in the length or periodicity of follow-up or ApoE E4 allele prevalence. Men were more highly educated (median 17 years compared to median 16 years for women, *p* < 0.001). As expected, due to lack of intracranial volume adjustment, women exhibited significantly lower baseline volumes of both CP and LV. Overall, 31 individuals had GDS =>5, indicating possible depression, however the overall level of depressive symptoms was low (median 1 point of 15). There was no statistically significant sex difference in history of hypertension, GDS or BMI.

The majority of subjects in the study (239/613, 39.0%) belonged to the stable MCI group, followed by the CN (188/613, 30.7%), convertor (98/613, 16.0%) and dementia (88/613, 14.4%) groups (Table 1B). In the convertor group, 24/98 (24.5%) belonged to the CN group at baseline, while 74/98 (75.5%) were part of the MCI group. There were significant between-diagnostic-group differences in almost all variables. Unsurprisingly due to expected overall brain atrophy, LV volume followed the order of progression NC < MCI < convertors < AD. Less predictably, but consistently with previous studies, similar progression was seen in baseline CP volume. Unsurprisingly due to the severity of their impairment, participants with dementia at baseline only attended a median of 3 follow-up visits over 1.07 years (as compared to 4 visits over 2.14–2.18 years for other groups). Diagnostic groups differed both in their mean BMI (lowest 25.74 kg/m^2^ for dementia, *p* = 0.004) and median GDS (highest 2.0 for MCI, *p* < 0.001).

All subsequent analyses were replicated with years of education as a variable of interest; since there were no significant effects, these results are not reported further.

### 3.2. Linear mixed effects models – CP change In time and basic model

Overall, we observed a statistically significant increase in CP volume over time: 14.92 mm^3^ per year, 95% CI (11.05, 18.77), *p* < 0.001 (Table 2). Due to the design of the model, the effect of the variable on CP volume should be considered as at baseline, unless follow-up time is introduced as an interaction. For categorical variables, all values should be interpreted as comparison to: male sex, CN diagnostic group, ApoE E4 non-carrier group and no hypertension group.

**Table 2:** Results of main linear mixed effects model.

Variable	Estimate	Std. Error	*p*-value	95% CI
(Intercept)	1119.73	230.91	<0.001	(670.78, 1568.72)
Time [Y]	14.92	1.97	<0.001	(11.05, 18.77)
Sex	−315.71	30.34	<0.001	(−374.71, −256.71)
Group: convertor	117.55	46.07	0.011	(28, 207.2)
Group: AD	187.27	46.84	<0.001	(96.32, 278.71)
Group: MCI	23.11	32.96	0.483	(−41.06, 87.27)
ApoE E4 heterozygote	14.31	32.17	0.657	(−48.27, 76.82)
ApoE E4 homozygote	4.11	52.94	0.938	(−98.9, 106.98)
Age at baseline [Y]	14.61	2.14	<0.001	(10.44, 18.77)
Education [Y]	3.84	5.7	0.501	(−7.24, 14.93)
Hypertension	20.02	29.93	0.504	(−38.16, 78.21)
Baseline BMI [kg/m^2^]	2.22	3.05	0.467	(−3.71, 8.14)
Baseline GDS	−4.35	9.82	0.658	(−23.45, 14.75)

Complete results of linear mixed model with CP volume as the dependent variable and random intercept with clustering by patient ID. Predictor variables included exactly as portrayed. Sex coded as 0 = males, 1 = females. Diagnostic groups as compared to stable no impairment group. ApoE E4 heterozygote and homozygote as compared to ApoE E4 non-carrier. Hypertension as compared to no hypertension. Rounding for 2 decimal places (3 for *p*-value). CI, confidence interval; Time, time from baseline in years (with baseline = 0); AD, Alzheimer’s disease dementia; MCI, mild cognitive impairment; BMI, body mass index; GDS, geriatric depression scale.

At baseline, both the AD and convertor groups exhibited a larger CP [187.27, 95% CI (96.32, 278.71) *p* < 0.001, and 117.55, 95% CI (28, 207.2), *p* = 0.011, respectively], while the MCI group showed no significant difference from the CN group. Other significant baseline effects were female sex [−315.71, 95% CI (−374.71, −256.71), *p* < 0.001] and baseline age [14.61, 95% CI (10.44, 18.77), *p* < 0.001].

### 3.3. Subgroup effects – sex

**Figure 1:**
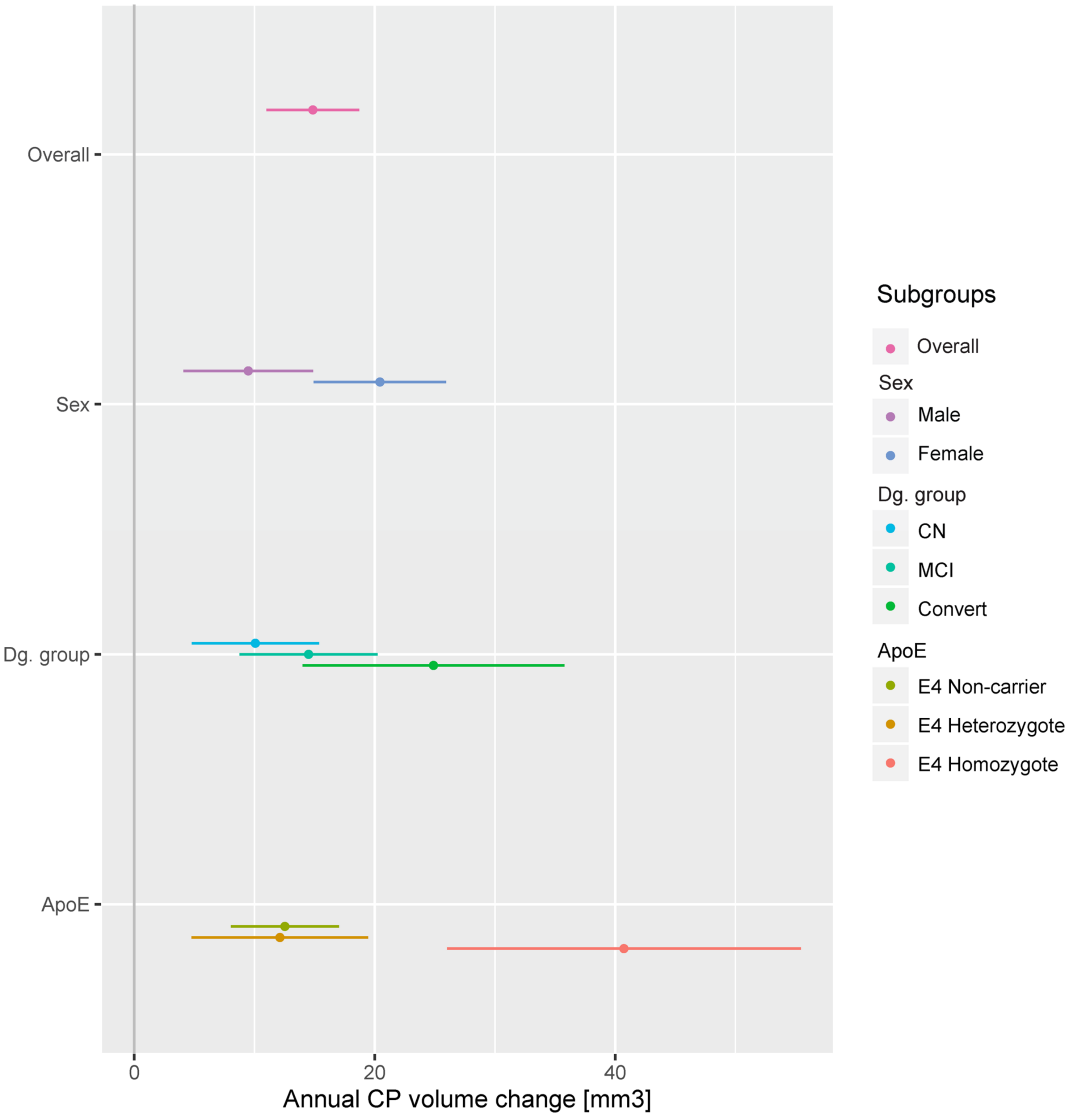
Annual CP change in selected subgroups. A dot and whiskers plot presenting the results of linear mixed effects models, representing the fixed effect of time in years on CP volumes, i.e. annual CP volume change. Models were fitted separately in all subgroups and adjusted for baseline effects of sex, baseline age, diagnostic group and ApoE as appropriate. Points represent temporal effect model estimates, while adjoining lines represent the confidence intervals. The stable dementia group (no significant change) was excluded for presentation purposes. CP, choroid plexus; CN, cognitively normal; MCI, mild cognitive impairment; Convert, conversion to MCI or dementia during follow-up.

**Figure 2:**
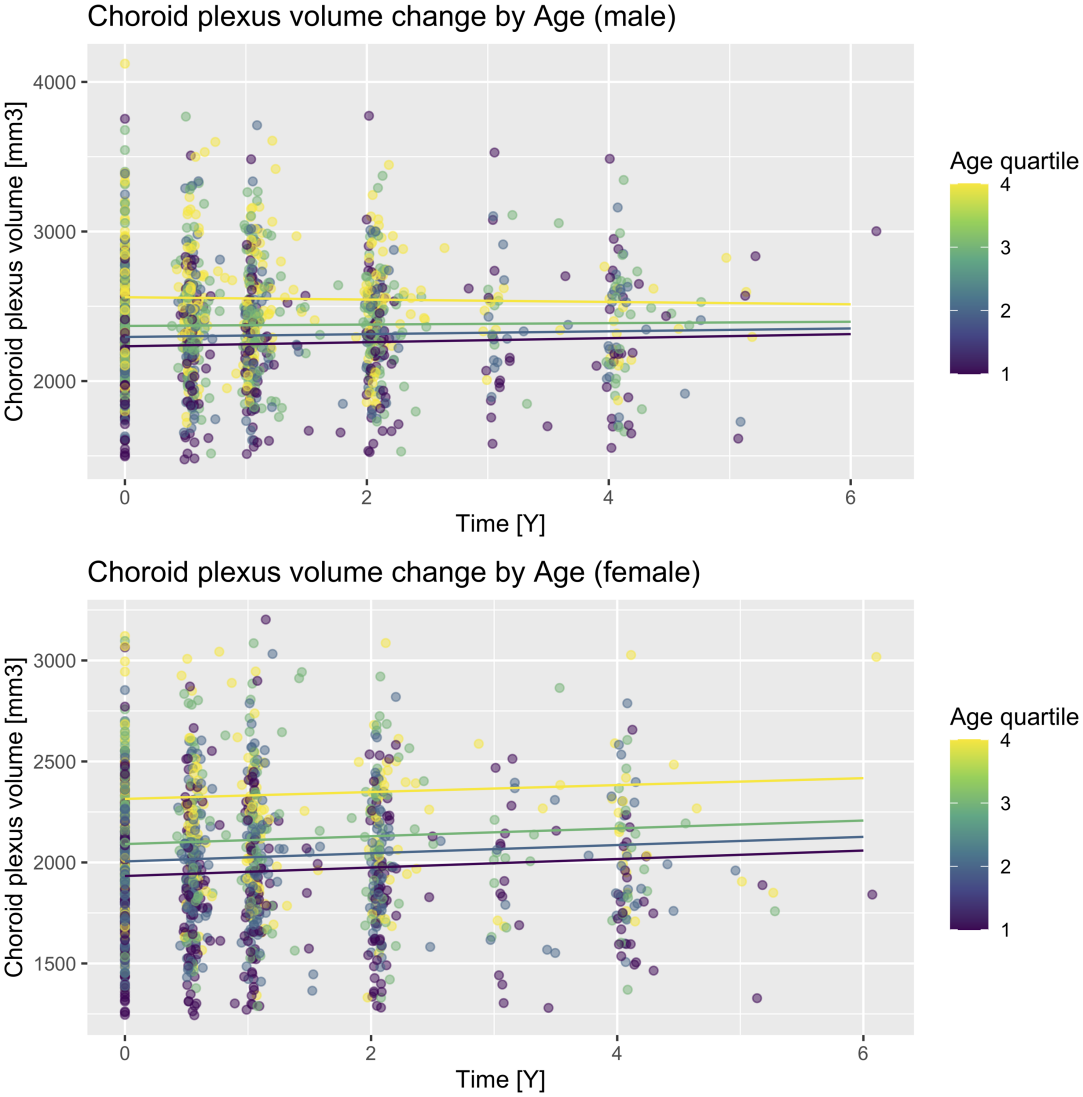
CP volume change according to sex and baseline age quartiles. A scatter plot with prediction lines of actual CP volume values and their change in time. Lines are predictions calculated for each follow-up time increment in each quartile and sex subgroup for ApoE E4 non-carriers and the cognitively unimpaired diagnostic subgroup. Quartiles are delineated as follows: Q1 (55.0–67.6), Q2 (67.6–72.1), Q3 (72.1–77.5), Q4 (77.5–91.4).

Annual CP volume enlargement appeared more than two times faster in females [20.43 mm^3^, 95% CI (14.91, 25.93), *p* < 0.001, Table 3A; Figures 1, 2] as compared to males [9.48 mm^3^, 95% CI (4.08, 14.87), *p* < 0.001], for the interaction (annual CP volume difference of females from males): 11.04, 95% CI (3.33, 18.74), *p* = 0.005. Models with other temporal interactions (diagnostic group, age, ApoE, education, hypertension, BMI, and GDS) did not dissipate the association, suggesting that this sex difference was not (fully) mediated by the effect of other temporal associations (Table 4).

**Table 3:** Results of subgroup analyses **(A)** and quantitative variable analyses **(B)** – temporal effects on choroid plexus.

Variable	Subgroup	Estimate	Std. Error	*p*-value	95% CI
**(A)**					
Sex	Female*	20.43	2.81	<0.001	(14.91, 25.93)
	Male*	9.48	2.75	<0.001	(4.08, 14.87)
Dg. Group	CN	10.07	2.7	<0.001	(4.77, 15.36)
	MCI	14.5	2.93	<0.001	(8.75, 20.23)
	AD	20.41	13.38	0.129	(−6.09, 46.52)
	Convertor*	24.88	5.56	<0.001	(14, 35.82)
ApoE	ApoE E4 non-carrier	12.52	2.29	<0.001	(8.02, 17.02)
	ApoE E4 heterozygote	12.12	3.75	0.001	(4.75, 19.45)
	ApoE E4 homozygote*	40.72	7.51	<0.001	(25.97, 55.46)
Hypertension	No	15.87	2.58	<0.001	(10.8, 20.93)
	Yes	13.57	3.03	<0.001	(7.62, 19.49)
**(B)**					
Age*		−0.6	0.27	0.027	(−1.14, −0.07)
Education		−1.14	0.75	0.131	(−2.61, 0.34)
BMI		−0.7	0.4	0.078	(−1.49, 0.08)
GDS*		2.79	1.33	0.036	(0.18, 5.4)

Annual change in choroid plexus volume in specified subgroup/for one unit of quantitative variable. Temporal effects (as obtained by time × variable interaction) only. Baseline corrected for age, and for sex, diagnostic group and ApoE as appropriate. Asterisk (*) denotes significance for the group difference (time × variable interaction on pooled data). All quantitative variables (Table 3B) considered as baseline values only. Sex coded as 0 = males, 1 = females. For multi-level variables Dg. group and ApoE, the comparison variable is CN and ApoE E4 non-carrier, respectively. Rounding for 2 decimal places (3 for *p*-value). CI, confidence interval; AD, Alzheimer’s disease dementia; MCI, mild cognitive impairment; BMI, body mass index; GDS, geriatric depression scale.

**Table 4:** Results of two-variable temporal effect analyses.

Model	Subgroup	Estimate	Std. Error	*p* value	95% CI
Sex × DG group	Sex (female)*	11.21	3.95	0.005	(3.48, 18.94)
	Convert*	14.75	5.72	0.01	(3.55, 25.97)
	AD	12.87	11.35	0.257	(−9.37, 35.07)
	MCI	5.47	4.44	0.218	(−3.23, 14.17)
Sex × ApoE	Sex (female)*	11.72	3.92	0.003	(4.04, 19.4)
	ApoE E4 heterozygote	−0.98	4.24	0.817	(−9.28, 7.32)
	ApoE E4 homozygote*	28.68	7.11	<0.001	(14.77, 42.6)
Sex × Age	Sex (female)*	10.44	3.94	0.008	(2.71, 18.15)
	Age*	−0.55	0.27	0.045	(−1.08, −0.01)
DG group × ApoE	Convert	10.19	5.98	0.089	(−1.52, 21.91)
	AD	5.65	11.59	0.626	(−17.07, 28.33)
	MCI	2.88	4.47	0.519	(−5.87, 11.63)
	ApoE E4 heterozygote	−1.64	4.34	0.705	(−10.14, 6.85)
	ApoE E4 homozygote*	24.53	7.48	0.001	(9.89, 39.16)
DG group × Age	Convert*	14.83	5.73	0.01	(3.62, 26.05)
	AD	12.92	11.37	0.256	(−9.36, 35.16)
	MCI	3.53	4.45	0.427	(−5.18, 12.24)
	Age*	−0.63	0.28	0.021	(−1.17, −0.1)
ApoE × Age	ApoE E4 heterozygote	−1.06	4.26	0.804	(−9.41, 7.29)
	ApoE E4 homozygote*	26.41	7.18	<0.001	(12.34, 40.48)
	Age	−0.49	0.28	0.076	(−1.03, 0.05)
GDS × DG group	Convert*	12.6	5.9	0.033	(1.07, 24.15)
	AD	9.4	11.41	0.41	(−12.97, 31.72)
	MCI	2.72	4.57	0.552	(−6.23, 11.66)
	GDS	2.16	1.39	0.119	(−0.55, 4.88)
GDS × Sex	Sex (female)*	10.74	3.93	0.006	(3.03, 18.43)
	GDS*	2.65	1.33	0.047	(0.04, 5.26)
GDS × ApoE	ApoE E4 heterozygote	0.21	4.24	0.96	(−8.1, 8.52)
	ApoE E4 homozygote*	27.88	7.12	<0.001	(13.94, 41.82)
	GDS*	2.62	1.33	0.049	(0.02, 5.23)
GDS × Age	Age	−0.52	0.28	0.06	(−1.06, 0.02)
	GDS	2.36	1.35	0.081	(−0.29, 5.01)

Temporal effects (as obtained by time × variable interaction) only, each variable × time interaction calculated separately in the same model. Baseline corrected for sex, diagnostic group, ApoE and baseline age as appropriate. Asterisk (*) denotes significant temporal effects (even with inclusion of the second variable’s temporal effect into the model). Sex coded as 0 = males, 1 = females. For multi-level variables Dg. Group and ApoE, the comparison variable is CN and ApoE E4 non-carrier, respectively. Rounding for 2 decimal places (3 for *p*-value). CI, confidence interval; AD, Alzheimer’s disease dementia; MCI, mild cognitive impairment; GDS, geriatric depression scale.

### 3.4. Subgroup effects – Diagnostic groups

We observed a significant annual enlargement of CP in all groups besides the stable dementia group [20.41, 95% CI (−6.09, 46.52), *p* = 0.129]. The largest increase was present in the convertors group [24.88, 95% CI (14, 35.82), *p* < 0.001] followed by the stable MCI group [14.5, 95% CI (8.75, 20.23), *p* < 0.001], with the smallest increase in the stable CN group [10.07, 95% CI (4.77, 15.36), *p* < 0.001, Table 3A, Figure 1; Supplementary Figure 1]. However, when compared to the CN group, only the convertors group showed a significant increase [14.75, 95% CI (3.52, 25.99), *p* = 0.010 for the interaction].

Exploring relationships with other temporal variables, we found that while sex, baseline age, hypertension, BMI or GDS did not appreciably diminish the convertors group difference from CN, inclusion of the ApoE temporal component appeared to fully dissipate its statistical significance [10.19, 95% CI (−1.52, 21.91), *p* = 0.089, Table 4]. This suggests a modifying relationship of ApoE on CP enlargement with progression of cognitive impairment.

### 3.5. Subgroup effects – ApoE

We assessed the CP longitudinal changes in three ApoE subgroups (ApoE E4 non-carriers, heterozygotes or homozygotes, Table 3A; Figure 1). All ApoE subgroups exhibited significant change in CP volume in time, which was comparable in the ApoE E4 non-carrier and heterozygote groups [12.52, 95% CI (8.02, 17.02), *p* < 0.001, and 12.12, 95% CI (4.75, 19.45), *p* = 0.001, respectively]. The increase in CP size in the ApoE E4 homozygote group was more than three times larger [40.72, 95% CI (25.97, 55.46), *p* < 0.001]. Only the ApoE E4 homozygote group was significantly different from the non-carrier group [28.15, 95% CI (14.2, 42.1), *p* < 0.001 for the interaction]. Other temporal variables did not appear to diminish the association (Table 4).

### 3.6. Variable effects – Baseline age

Baseline age exhibited a quantitatively relatively modest, but statistically significant negative effect on CP volume [−0.6, 95% CI (−1.14 to −0.07), *p* = 0.027]. Inclusion of ApoE or GDS temporal effect into the model rendered the CP-age association insignificant [e.g., −0.49, 95% CI (−1.03, 0.05), *p* = 0.076 for the model including ApoE, Table 4]; other temporal associations did not (fully) reduce the effect.

We then fitted similar models in both sex subgroups: for males, the CP-age association was statistically significant [−0.90, 95% CI (−1.64, −0.16), *p* = 0.018], while for females, it was not [−0.16, 95% CI (−0.94, 0.61), *p* = 0.681]. Next, we calculated predicted CP volume at each time increment of each baseline age quartile (Figure 2). While on visual inspection, a sex difference can be seen for all quartiles, it is most evident for the fourth quartile (age range 77.5–91.4). This was confirmed in a separate model in the fourth quartile subgroup, including both sexes (CP volume as dependent variable, time, diagnostic group, ApoE, sex, sex × time interaction), where the sex difference in longitudinal CP enlargement was the most prominent [statistically non-significant increase in CP volume for males, *p* = 0.627, and additional 20.39 mm^3^ for females, 95% CI (1.59, 39.17), *p* = 0.034].

### 3.7. Variable effects – Hypertension, BMI, depression

Hypertension and BMI were not associated with a change in CP volume in any of the univariate models (Tables 3A,B; Figure 1). Higher baseline level of depressive symptoms (GDS) was associated with an increase in CP volume: 2.79, 95% CI (0.18, 5.4), *p* = 0.036, which can be interpreted as an additional annual increase in CP volume of 2.79 mm^3^ for every additional point on the GDS scale. This association was rendered not significant in models with diagnostic group and baseline age (Table 4), suggesting that the increase of CP volume with depressive symptoms could be a by-product of the associations of CP volume and either diagnostic group or baseline age.

### 3.8. Lateral ventricular volume

We repeated the same basic analyses using log-transformed lateral ventricular volume to determine if ventricular volume increase in time exhibited similar relationships. LV volume enlarged overall [0.05, 95% CI (0.05, 0.05), *p* <0.001, Supplementary Figure 2], and was significantly larger at baseline in all diagnostic groups as compared to CN. The diagnostic group differences appeared similar in size and direction to the effects of time on CP volumes, with a progressive annual increase CN < MCI < convertor < AD. However, in contrast to CP enlargement, LV volume progression when compared to the CN group was significant for all cognitively impaired subgroups (*p* s < 0.001 to 0.001 for the comparison, Supplementary Table 1). There was no statistically significant sex difference in progression in time. In contrast to our CP results, where CP increased significantly faster in the ApoE E4 homozygote group, LV volume increase with ApoE seemed to exhibit a dose-dependent relationship to ApoE (Supplementary Table 1).

Lastly, we tested a model with CP as a response variable, time, LV volume and its time interaction. While the results showed a statistically significant temporal effect of LV volume × time interaction (*p* < 0.001), the CP temporal effect remained significant (*p* < 0.001) and was not (fully) diminished, suggesting a degree of independence from LV.

## 4. Discussion

In this pilot study, we assessed macrostructural CP changes in a large cohort spanning the spectrum of cognitive impairment in the context of AD. This is, to our knowledge, the first study to look at longitudinal associations between CP volume, sex and cognitive impairment. We found an overall increase in CP volume across diagnostic, sex and ApoE groups. In addition, we found significant differences in CP enlargement by diagnostic groups (CN < MCI < convertor progression, with the convertor subgroup differing from the CN subgroup), by ApoE (more than three times larger in the ApoE E4 homozygote as compared to non-carrier or heterozygote groups), and by sex (more than twice as large in females as compared to males). More minute effects on CP volume were also observed in analyses with baseline age (negative association) and GDS, a depressive symptom proxy (positive association).

First, our finding of overall annual choroid plexus enlargement was novel in the context of neurodegenerative diseases. The volume increase was in contrast to previous longitudinal studies on CP volume in stroke and multiple sclerosis (30, 32), where no change in time was observed. However, this could be attributed to both differing study population as well as lower number of subjects and shorter follow-up in the cited studies. Another explanation could be neurodegeneration-specific mechanisms of CP enlargement.

This hypothesis was further explored by considering diagnostic group × CP volume associations. In agreement with previous studies (35–37), we found larger CP volume at baseline in subjects with dementia, as compared to stable CN (Tables 1B, 2). However, due to our longitudinal study design, we were able to additionally include a group of convertors, which also exhibited a baseline difference from CN, showing further CP increase with cognitive impairment. Longitudinal associations further corroborated the disease and CP volume progression relationship by showing a novel finding of independent CP volume growth with increasing impairment (CN < MCI < convertor). In addition, since solely the convertors group exhibited longitudinal growth as compared to CN group, CP enlargement may be linked to progression of cognitive impairment.

Interestingly, the relationship between choroid plexus enlargement and disease progression appeared to be modified by the temporal effects of ApoE E4 homozygote status, suggesting potential ApoE-related mechanisms. A previous cross-sectional study exploring ApoE effects on CP volume (37) found no significant effect; this was, however also true for our analysis (Table 2), which only revealed the ApoE effect in temporal associations. Longitudinal associations between ApoE and CP enlargement comparable to our analysis have previously not been described and warrant further exploration in the future.

Second, in our study, we specifically aimed to assess sex effects due to potential sex-differing CP pathophysiology. Sex differences in CP volume have previously been explored in cross-sectional associations (25, 31, 37). While two of the studies on healthy ageing and multiple sclerosis did not find an appreciable sex difference, the third study on Alzheimer’s disease population described larger CP volumes in males (37). In a cross-sectional comparison (without intracranial volume correction) we found a similar male predilection in CP volume (Table 1A). We confirmed the direction correcting for estimated intracranial volume using the same method as the cited study. While past evidence of sex-differing CP mechanisms supports existence of a sex difference in CP volume (13, 25, 50), this could also be a reflection of the used intracranial volume correction method (39) and warrants further exploration using other alternative correction methods. Longitudinal within-subjects comparisons, such as in our study, do not require intracranial volume correction, and therefore eliminate this source of variability. The lack of a sex difference in the two previous studies could, however, be caused by smaller sample sizes, because exploration of sex differences generally requires larger sample sizes than study of main effects (51). If a cross-sectional sex difference is confirmed, potential reasons for the discrepancy between larger male volume at baseline and faster female CP enlargement in time could include diverging mechanisms of CP enlargement. Studies have described faster cognitive progression in women as compared to men, or higher probability of conversion from MCI to dementia (52, 53). While specific mechanisms for this are unknown, progressive CP enlargement could be caused by similar processes. We explore the potential mechanisms below.

Some surprising results were obtained in analyses with baseline age. Baseline CP volume was significantly increased in older subjects (14.61 mm^3^ for every additional year of baseline age, section 3.1 and Table 2), indicating that before entry to the study, older subjects tended to have higher CP volumes, which was in agreement with previous studies (25). However, longitudinal analysis showed a small but statistically significant negative association between CP volume in time and advancing age – though it must be noted that this effect never translated to a decrease in CP volume, rather an absence of its increase. This effect appeared strongest in the male subgroup, and especially in its oldest quartile (Figure 2). However, this effect was rendered not significant when either temporal effects of ApoE or GDS were included in the analysis. This could indicate that the age association is a by-product of the stronger association of CP longitudinal volume increase and ApoE (or GDS). However, the sex difference also appeared strongest in the highest age quartile: this could be a sign of a ceiling effect on CP volume, which might not get enlarged further after a certain volume threshold. Potential mechanisms for the age and sex difference are described below.

In addition to the variables discussed above, we assessed the longitudinal relationship between changes in CP volume and hypertension, BMI (body adiposity proxy) and GDS (depression proxy). While we (unsurprisingly due to overall low levels of depressive symptoms) did not find any cross-sectional associations between GDS and CP volume, we observed an increase in CP volume in time with increasing GDS. Since, to our knowledge, longitudinal associations of CP and depressive symptoms were not explored before, this represents a novel result. It is, however, consistent with a previous study that established the link between CP volume, neuroinflammation and depression (29).

Surprisingly, we did not find any significant associations (cross-sectional or longitudinal) with either hypertension or BMI, in contrast to previous cross-sectional studies (34, 37). For obesity, Alisch et al. (34) found significant associations between CP volume and BMI in cognitively healthy subjects across a wide age-span (22–94), and in relatively lean individuals (BMI 25.8). The lack of significance in our study could be caused by population differences, since the population in our study is overall older, with higher BMI, and with cognitive impairment. Similar to the negative temporal association of age and CP volume, this could also represent a ceiling effect, where after reaching a certain threshold, the effect of BMI on CP volume diminishes. Choi et al. (37) showed increased volume in Korean hypertensive individuals (in contrast to our results), however the associations with age, sex, and diagnostic group were consistent with our study. Therefore, the relationship of BMI and hypertension to CP volume should be explored in future studies with diverse populations to ascertain the nature of the association.

Choroid plexus enlargement could overall be attributed to several different mechanisms. First, choroid plexus enlargement could be due to neuroinflammation. Choroid plexus volume was found to be associated with active neuroinflammation (7) in both predominantly inflammatory diseases such as multiple sclerosis (31) and other diseases linked with neuroinflammation such as depression (29). In fact, in an animal model of multiple sclerosis, CP volume increase was specifically associated with microglial infiltration and activation, astrocyte activation (linked to white matter remodeling), increased albumin content in the CSF and increased expression of genes related to T cell adhesion, differentiation and activation (7). This suggests that CP enlargement could be due to increased infiltration by both CNS resident cells (microglia) and peripheral immune cells (T cells, and by extension macrophages), as well as increased barrier permeability, oxidative stress and cellular edema.

CP-mediated neuroinflammation has been suggested as a possible pathophysiological mechanism of AD and other neurodegenerative diseases (11, 12) and corroborated by reversal of AD-related cognitive decline and beta-amyloid deposition after introduction of CP targeted immune-suppression (38). CP inflammation was also linked to increased CP volume and remodeling in AD (36). Although sex differences in CP immunologic activity in cognitive impairment have to our knowledge not been directly explored, immune mechanisms in AD show significant and complex interactions with sex, with generally, but not exclusively, higher pro-inflammatory activity in females (18, 22, 23, 54, 55). In fact, a recent study noted increased activation of microglia, the major cell type present in inflamed CP, in reaction to beta amyloid deposition in females (56). Interestingly, in the same study, microglial activation was attenuated by ApoE deletion independent of sex, suggesting independent effects of ApoE and female sex on neuroinflammation.

In this context, the enlargement of CP volume with female sex, cognitive impairment and ApoE E4 could represent a specific, yet largely independent, pro-inflammatory signature. This could present sex- and impairment-specific hypotheses for future *in vitro* and *in vivo* CP inflammation studies, as well as an argument for sex and ApoE stratification in clinical trials of immune-active agents.

The relationship between sex, age and CP volume could also be caused by immunologic factors. Immunosenescence, represented by a decrease in both T-cell and B-cell counts, as well as T-cell proliferative activity, is accelerated in males compared to females (57). Thus, comparatively lower increase in CP volumes could be linked to lessened CP immunologic activity in males. Alternative explanations include sex-divergent CP microstructural changes with age or a non-linear relationship between CP volume and age. Sex divergent CP structure changes have been described, e.g., in the case of calcifications (58). In terms of age and CP relationship, women were younger in terms of our sample, but they also tend to be younger in metabolic age (59), telomere length (60), and complex differences in metabolism including mitochondrial function (61). Thus, the negative longitudinal association between CP volume and age in men could be a sign of a ceiling effect: lack in CP increase after a certain volume threshold (which could possibly occur around 77.5–91.4 years of age for men, as is our last quartile). Further exploration of these findings is warranted.

In the context of Alzheimer’s disease, choroid plexus has been described to undergo other changes which could contribute to its increased volume, such as basal membrane thickening and complex remodeling including beta amyloid deposition (11, 15, 36, 62). ApoE accumulation in CP in the form of lipofuscin or complex filamentous structures with lipid droplets has also been described (63), providing another potential link between ApoE E4 and CP enlargement. Other hypotheses for CP enlargement in time could involve degenerative processes implicated in age-related increase in CP size, such as cellular degeneration, stroma thickening, psammoma body accumulation and calcifications (58, 64). CP enlargement could also be related to adaptive growth factor upregulation (65–67), although this relationship could again be linked to the inflammatory cascade (68). Compromise of blood-CSF barrier could also be involved, however increased CP volume was linked to an increase in CSF albumin (7), while the CSF/blood albumin ratio is generally noted to be increased in males (69, 70), and therefore likely constitutes a more complex relationship.

Overall, the observed differences in CP volume, including sex, diagnostic group, ApoE genotype, and age differences likely represent a complex web of immunologic and metabolic factors, which could be downstream effects of shifting hormonal landscapes (71), past sex hormone exposures (72), or sex differences on a sub/cellular (61) or chromosomal level (73).

Increased cross-sectional CP volume has been suggested as a potential biomarker for AD due to its independent relationship to MMSE (37). Our results suggest that an increase of the CP volume in time could be linked to the progression of the disease (when compared to subjects with normal cognition, this association appears linked to CP volume enlargement associated with ApoE). However, CP volume increase is non-specific due to its link (both cross-sectionally and longitudinally) with a number of other neuropsychiatric or systemic diseases, as described above. As such, CP volume increase does not follow established criteria of a diagnostic or prognostic biomarker (74). Rather, we believe its value lies elsewhere: further investigation of CP-related mechanisms of AD could help disentangle some pathophysiologic underpinnings of this complex disease, in particular with regards to the role of neuroimmune interactions. In addition, this line of research could provide potential targets for future interventions.

It has to be considered that our results could be affected by ventriculomegaly, either through apparent increase in CP volume caused by reduced pressure in the enlarged ventricles, or through overestimation of CP segmentation due to CSF flow artifacts (35). To test this hypothesis, we replicated basic analyses using log-transformed LV volume as the response variable, and tested a model with both the CP and LV temporal relationships. Since CP temporal effects remained significant even after inclusion of LV, they are apparently associated but CP exhibits independent temporal effects. Additionally, LV volumes showed no appreciable sex difference and exhibited a dose-dependent ApoE E4 relationship, suggesting that the sex and ApoE E4 homozygote effects could be unique to the CP.

### 4.1. Strengths and limitations

Our study included a large sample of subjects (*n* = 613) with a median of four follow-up points. Longitudinal design and chosen statistic methods minimized interference from inter-individual variability in unrelated variables such as intracranial volume. Despite this, our study also has limitations. Although the FreeSurfer automatic software has been used for CP segmentation in several preceding studies, it is likely not as accurate as other methods, such as manual segmentation, or machine learning approaches (33, 37, 75). Our findings should therefore be confirmed with more robust segmentation methods. Despite the overall size of the dataset, some groups were relatively under-represented (convertors, AD dementia), which could limit the ability to find associations specific to those diagnoses. Additionally, the overall length of follow-up was relatively short, with a median of 2.10 years. Longer follow-up would enable more consistent evaluation of CP changes, as well as possibly capture individuals wrongly present in the “stable MCI” or “stable CN” rather than the “convertors” group due to slower conversion process. Due to including clinically and not biomarker-defined cognitive impairment populations, our results cannot be generalized to Alzheimer’s disease etiology and should rather be interpreted as mild cognitive impairment or dementia without evidence of non-Alzheimer’s pathology. Lastly, we did not perform a multiple comparisons adjustment due to the complexity of the presented models. While this presents a potential risk for false-positive results, we believe this risk to be relatively low due to generally high degrees of statistical significance. Overall, the results of this pilot study should be confirmed in future studies.

## 5. Conclusion

Our results contribute to the body of knowledge on sex differences in AD with a novel finding of twice the annual choroid plexus enlargement in females, and provide putative support for CP-mediated mechanisms in the progression of AD, its sex difference, and potential ApoE E4-related mechanisms.

## Data availability statement

Publicly available datasets were analyzed in this study. This data can be found at: http://adni.loni.usc.edu/.

## Ethics statement

The studies involving human participants were reviewed and approved by ethics committees/institutional review boards of all ADNI participating sites. A full list is available at https://adni.loni.usc.edu/. The patients/participants provided their written informed consent to participate in the ADNI study.

## Author contributions

JN conceptualized the study, organized the database, conducted the statistical analysis and wrote the manuscript. MF contributed to interpretation, manuscript writing and critical reading. AF consulted on statistical analyses, conceptualization and interpretation and contributed by critical reading of the manuscript. OL, VM, and JS contributed to interpretation, critical reading and feedback on the analyses and the manuscript. JH contributed to the conceptualization of the study, feedback on the analyses and critical reading of the manuscript. All authors contributed to the article and approved the submitted version.

## Funding

This work was supported by the Charles University Grant Agency (GA UK) project no. 436119 at Charles University, Second Faculty of Medicine. Supported by project no. LX22NPO5107 (MEYS): Financed by EU – Next Generation EU. Data collection and sharing for this project was funded by the Alzheimer’s Disease Neuroimaging Initiative (ADNI) (National Institutes of Health Grant U01 AG024904) and DOD ADNI (Department of Defense award number W81XWH-12-2-0012). ADNI is funded by the National Institute on Aging, the National Institute of Biomedical Imaging and Bioengineering, and through generous contributions from the following: AbbVie, Alzheimer’s Association; Alzheimer’s Drug Discovery Foundation; Araclon Biotech; BioClinica, Inc.; Biogen; Bristol-Myers Squibb Company; CereSpir, Inc.; Cogstate; Eisai Inc.; Elan Pharmaceuticals, Inc.; Eli Lilly and Company; EuroImmun; F. Hoffmann-La Roche Ltd. and its affiliated company Genentech, Inc.; Fujirebio; GE Healthcare; IXICO Ltd.; Janssen Alzheimer Immunotherapy Research & Development, LLC.; Johnson & Johnson Pharmaceutical Research & Development LLC.; Lumosity; Lundbeck; Merck & Co., Inc.; Meso Scale Diagnostics, LLC.; NeuroRx Research; Neurotrack Technologies; Novartis Pharmaceuticals Corporation; Pfizer Inc.; Piramal Imaging; Servier; Takeda Pharmaceutical Company; and Transition Therapeutics. The Canadian Institutes of Health Research is providing funds to support ADNI clinical sites in Canada. Private sector contributions are facilitated by the Foundation for the National Institutes of Health (www.fnih.org). The grantee organization is the Northern California Institute for Research and Education, and the study is coordinated by the Alzheimer’s Therapeutic Research Institute at the University of Southern California. ADNI data are disseminated by the Laboratory for Neuro Imaging at the University of Southern California.

## Conflict of interest

MF is the Cofounder and CSO of the Women’s Brain Project. She has received personal fees for consulting from Lilly, Roche, and Lundbeck, not related to the present project. AF was employed by Women’s Brain Project. JH received consultancy and lecture fees from Biogen, Schwabe, Zentiva and holds share options in Alzheon company.

The remaining authors declare that the research was conducted in the absence of any commercial or financial relationships that could be construed as a potential conflict of interest.

## Publisher’s note

All claims expressed in this article are solely those of the authors and do not necessarily represent those of their affiliated organizations, or those of the publisher, the editors and the reviewers. Any product that may be evaluated in this article, or claim that may be made by its manufacturer, is not guaranteed or endorsed by the publisher.

## Footnotes

^1^Available in full at http://adni.loni.usc.edu/methods/mri-tool/mri-analysis/

^2^ http://surfer.nmr.mgh.harvard.edu/

## Abbreviations

CP: Choroid plexus
ADNI: Alzheimer’s Disease Neuroimaging Initiative
CN: Cognitively normal
MCI: Mild cognitive impairment
AD: Alzheimer’s disease dementia
CSF: Cerebrospinal fluid
MRI: Magnetic resonance imaging
PET: Positron emission tomography
MMSE: Mini mental state exam
CDR: Clinical dementia rating
LV: Lateral ventricles
ANOVA: One-way analysis of variance
CI: Confidence interval
