# Supplementary material for: Longitudinal progression of choroid plexus enlargement is associated with female sex, cognitive decline and ApoE E4 homozygote status
Source: Front Psychiatry. 2023 Mar 8;14:1039239. doi: 10.3389/fpsyt.2023.1039239 (PMC10031049; doi:10.3389/fpsyt.2023.1039239)
Supplement: Supplementary file 1 [file Data_Sheet_1.docx]

Supplementary Material

# Supplementary Table 1

| Variable | Subgroup | Estimate | Std. Error | p-value | 95% CI |
| --- | --- | --- | --- | --- | --- |
| Dg. Group | CN | 0.04 | 0 | <0.001 | (0.04, 0.04) |
|  | MCI* | 0.04 | 0 | <0.001 | (0.04, 0.05) |
|  | AD* | 0.09 | 0 | <0.001 | (0.08, 0.1) |
|  | Convert* | 0.08 | 0 | <0.001 | (0.08, 0.09) |
| Sex | Female | 0.05 | 0 | <0.001 | (0.05, 0.05) |
|  | Male | 0.05 | 0 | <0.001 | (0.05, 0.05) |
| ApoE | E4 non-carrier | 0.04 | 0 | <0.001 | (0.04, 0.04) |
|  | E4 heterozygote* | 0.06 | 0 | <0.001 | (0.05, 0.06) |
|  | E4 homozygote* | 0.09 | 0 | <0.001 | (0.08, 0.09) |

**Supp. Table 1: Results of subgroup analyses – temporal effects on lateral ventricles (log-transformed).** Temporal effects (as obtained by time x variable interaction) only. Baseline corrected for sex, diagnostic group and ApoE as appropriate. Asterisk (*) denotes significance for the within-group difference (time*variable interaction on pooled data). Sex coded as 0 = males, 1 = females. For multi-level variables Dg. group and ApoE, the comparison variable is CN and ApoE E4 non-carrier, respectively. Rounding for 2 decimal places (3 for p-value). CI = confidence interval, AD = Alzheimer’s disease dementia, MCI = mild cognitive impairment.

# Supplementary Figure 1

**Supp. Figure 1:** **Choroid plexus volume change by diagnostic group.** The lines represent fixed effects (time) from linear mixed effects models with random intercept clustered by patient. Models were unadjusted. Points indicate individual datapoints. CP = choroid plexus, Y = years.

# Supplementary Figure 2


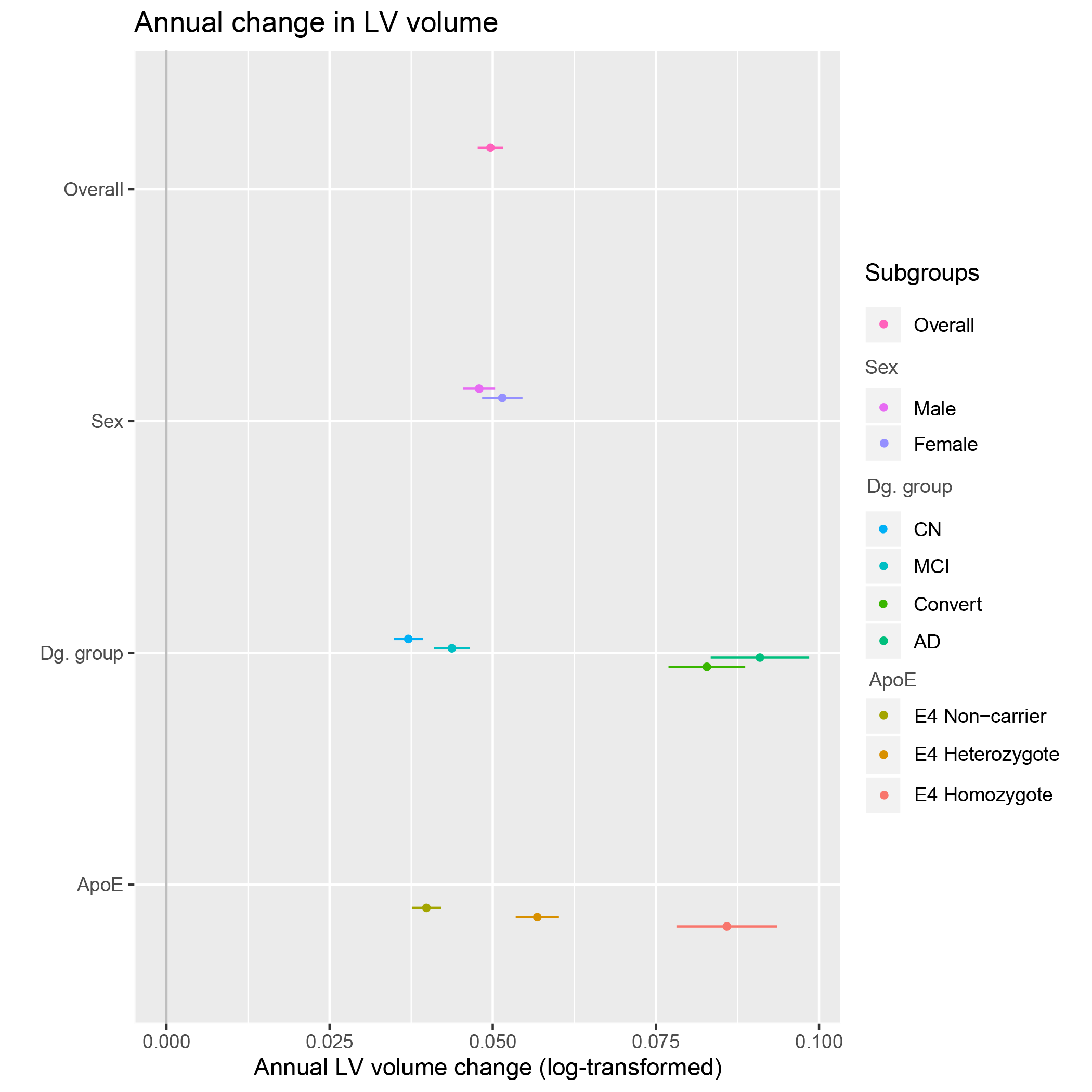


**Supp. Figure 2: Annual change in LV volume in subgroups.** A box and whiskers plot presenting the results of linear mixed effects models, representing the fixed effect of time in years on LV volumes (log-transformed due to left skew). Models were fitted separately in all subgroups and adjusted for baseline effects of sex, baseline age, diagnostic group, ApoE and education as appropriate. CP = choroid plexus, CN = cognitively normal, MCI = mild cognitive impairment, Convert = conversion to MCI or dementia during follow-up.
